# Local hypocretin-1 modulates terminal dopamine concentration in the nucleus accumbens shell

**DOI:** 10.3389/fnbeh.2012.00082

**Published:** 2012-11-28

**Authors:** Robin Patyal, Evan Y. Woo, Stephanie L. Borgland

**Affiliations:** Department of Anesthesiology, Pharmacology and Therapeutics, The University of British ColumbiaVancouver, BC, Canada

**Keywords:** hypocretin, orexin, dopamine, nucleus accumbens shell, voltammetry, glutamate

## Abstract

Hypocretins (hcrt), also known as orexins, play a critical role in reward-seeking behavior for natural rewards and drugs of abuse. The mesolimbic dopamine pathway that projects from the ventral tegmental area (VTA) to the nucleus accumbens (NAc) is critically involved in the neural mechanisms underlying reward-seeking and motivation. Hcrt immunopositive fibers densely project to the shell of the nucleus accumbens (NAcSh), suggesting that the NAcSh might be a site for the interaction between hcrt and dopaminergic modulation of reward-seeking behavior. While it is known that hcrt action in the VTA can increase dopamine in the NAc, it has not been determined if hcrt released locally at dopaminergic terminals in the NAcSh can modulate dopamine concentration. Here, we use fast scan cyclic voltammetry (FSCV) in forebrain slices containing the NAcSh to determine whether hcrt can alter evoked dopamine concentration. We found bath application of hcrt-1 increases phasically evoked dopamine release, without altering reuptake at dopamine terminals in the NAcSh. Hcrt-1-induced potentiation of dopamine concentration was inhibited by SB334867, a hcrt receptor 1 antagonist, as well as ionotropic glutamate receptor antagonists, AP-5, CNQX and DNQX. Taken together, these results suggest that local hcrt-1 can modulate dopamine in the NAcSh and may play a role in reward-seeking and appetitive behaviors.

## Introduction

The hypocretins (hcrt), also known as orexins, are neuropeptides produced solely in the lateral hypothalamic and perifornical areas (de Lecea et al., 1998; Sakurai et al., 1998). They are comprised of two distinct peptides; hypocretin-1 (hcrt-1) and hypocretin-2 (hcrt-2). Hypocretins activate two known G-protein coupled receptors, hcrt receptor 1 (hcrt-R1) and hcrt receptor 2 (hcrt-R2). Hypocretin neurons project locally within the hypothalamus and widely throughout the brain, suggesting hypocretin involvement in multiple physiological functions. Indeed, hypocretin has been implicated in sleep and wakefulness, energy homeostasis and addiction (Aston-Jones et al., 2009; Boutrel et al., 2010).

Dopamine neurons of the ventral tegmental area (VTA) project to the nucleus accumbens (NAc) via the medial forebrain bundle (Nauta et al., 1978). Tonic and phasic activity of dopamine neurons can promote dopamine release in NAc (Tsai et al., 2009). Phasic firing typically accompanies reward-predicting cues leading to reward seeking behaviors (Phillips et al., 2003; Roitman et al., 2004; Day et al., 2007; Tsai et al., 2009). The NAc receives excitatory glutamatergic inputs primarily from cortical and limbic structures, including the hippocampus, basolateral amygdala, prefrontal cortex, and various thalamic nuclei (Sesack et al., 1989; Berendse and Groenewegen, 1990; Callaway et al., 1991; Pennartz et al., 1994; Finch, 1996). While glutamatergic and dopaminergic inputs converge on the same MSNs, direct synaptic contacts between dopaminergic and glutamatergic axon terminals have not been observed in the NAc (Sesack and Pickel, 1990, 1992; O'Donnell and Grace, 1995; Mulder et al., 1998). The NAc consists of two anatomically, biochemically, and behaviorally distinct subregions referred to as the shell (NAcSh) and the core (Meredith et al., 1992, 1993, 2008; Sesack and Grace, 2010). Hypocretin neurons make dense projections to both dopamine neurons of the VTA as well as the medial NAcSh (Peyron et al., 1998; Baldo et al., 2003) where both hcrt-R1 and R2 are expressed (Marcus et al., 2001; Martin et al., 2002).

Many reports have demonstrated that hcrt-1 signaling in the VTA promotes reward-seeking behaviors, including reinstatement of extinguished drug seeking behavior (Harris et al., 2005; Wang et al., 2009; James et al., 2011), behavioral sensitization to cocaine (Borgland et al., 2006) or effort for sucrose, high fat or cocaine (España et al., 2010; Thompson and Borgland, 2011). Hcrt-1 increases dopamine neuronal firing (Korotkova et al., 2003; Muschamp et al., 2007; Moorman and Aston-Jones, 2010) and potentiates excitatory synaptic transmission onto dopamine neurons in the VTA (Borgland et al., 2006, 2009), a mechanism thought to promote arousal for salient events (Borgland et al., 2009; Thompson and Borgland, 2011). Hcrt-1 action in the NAcSh inhibits N-methyl-D-aspartate (NMDA)-induced currents in isolated medium neurons (MSNs; Martin et al., 2002). However, other reports suggest that hcrt-1 or hcrt-2 dose-dependently depolarizes (Mukai et al., 2009) and increases firing rate of MSNs in slices containing the NAcSh (Mukai et al., 2009; Mori et al., 2011). Hcrt-1 administered directly to the NAcSh promotes feeding and locomotor activity (Thorpe and Kotz, 2005) as well as dopamine-dependent turning behavior (Kotani et al., 2008).

Hcrt-1 in the VTA increases dopamine release in the NAc and prefrontal cortex (Narita et al., 2006; Vittoz and Berridge, 2006; Vittoz et al., 2008; España et al., 2011). Moreover, hcrt-1 in the VTA enhances cocaine-induced dopamine release (España et al., 2011). However, it is unknown if hcrt-1 within the NAcSh can modulate extracellular dopamine concentration [DA]_o_. Here, we tested the hypothesis that hcrt-1 increases evoked [DA]_o_ in the NAcSh using Fast Scan Cyclic Voltammetry (FSCV) at single pulse or phasic burst-like stimulation frequencies.

## Materials and methods

### Animals

All protocols were in accordance with the ethical guidelines established by the Canadian Council for Animal Care and were approved by the University of British Columbia Animal Care Committee. C57BL/6J mice were obtained from the University of British Columbia breeding facility.

### Slice preparation

Male C57BL/2J mice (25–30 g) were anesthetized with halothane or isoflurane, decapitated, and brains were extracted and prepared for slicing. Coronal slices (250 μm) containing the NAcSh were cut in ice-cold sucrose-containing artificial cerebrospinal fluid (aCSF) solution ([mM]: 87 NaCl, 2.5 KCl, 1.25 NaH_2_PO_4_, 25 NaHCO_3_, 7 MgCl_2_, 0.95 CaCl_2_, 75 sucrose) saturated with 95% O_2_/5% CO_2_ using a vibratome (Leica, Nussloch, Germany). Slices were incubated in warm (31.5°C) 95% O_2_/5% CO_2_ oxygenated aCSF ([mM]: 119 NaCl, 1.6 KCl, 1.0 NaH_2_PO_4_, 26.2 NaHCO_3_, 1.3 MgCl_2_, 2.5 CaCl_2_, 11 glucose; pH = 7.4) for at least 1 h and transferred to a recording chamber that was constantly perfused (gravity flow: 1.5 ml/min) with oxygenated aCSF (30–32°C). The slices were allowed to equilibrate in the recording chamber with the superfusion medium for an additional 20–30 min before experimentation.

### Fast-scan cyclic voltammetry

Evoked [DA]_o_ was measured using FSCV with carbon-fiber microelectrodes. Carbon fibers (7 μm diameter; Goodfellow) were pulled in glass electrodes and cut to a final exposed length of ~150 μm. Triangular waveforms (holding at −0.4 V) at 10 Hz (−0.4 to 1.0 V vs. Ag/AgCl at 400 V/s scan rate) were used. Catecholamine release was evoked using electrical stimulation applied with a bipolar stimulating electrode positioned flush with the tissue for local surface stimulation. To evoke [DA]_o_ in the NAcSh, either a single pulse was delivered or 100 Hz, 5 pulses to mimic phasic dopamine release (Rice and Cragg, 2004). The voltammetric electrode was positioned between the tips with the aid of a binocular microscope, and then lowered 50–100 μm into the tissue. Dopamine was identified by characteristic oxidation and reduction peak potentials (approx. +600 and −200 mV vs. Ag/AgCl). To determine the time course of dopamine, the current at the peak oxidation was plotted against time. Relative electrode sensitivities for dopamine were determined by obtaining voltammograms from exogenous application of dopamine (0.1–1 μM) made from stock solutions in 0.1 M HClO_4_ immediately before use.

### Drugs

Hcrt-1 or AP-5 was purchased from Tocris (Ellisville), reconstituted in ddH_2_0 and stored in aliquots at −20°C. Ten minutes prior to drug application aliquots were thawed and diluted to the working concentration in aCSF buffer. CNQX or DNQX was purchased from Sigma (Oakville) reconstituted in DMSO and stored in aliquots, protected from light, at −20°C. Prior to the experiment, these compounds were diluted in aCSF and used at 1/1000 working concentrations of DMSO. SB334867 was purchased from Tocris (Ellisville) reconstituted in DMSO and stored in aliquots at −20°C. Prior to the experiment, SB334867 was diluted in aCSF and used at 1/1000 working concentrations of DMSO. Our previous work has demonstrated that 1/1000 DMSO does not influence evoked [DA]_o_ (Mebel et al., 2012).

### Statistics

Values listed are means ± SEM. Statistical significance was assessed using a paired student *t*-test comparing a time-point on the baseline prior to drug application to a time-point after drug application. For multiple comparisons, One-Way ANOVA was used unless otherwise indicated. A difference of *p* < 0.05 was considered significant. Statistical tests were performed with GraphPad Prism v.5.

## Results

### Hcrt-1 does not modify single pulse evoked [DA]_o_ at terminals

**Figure 1:**
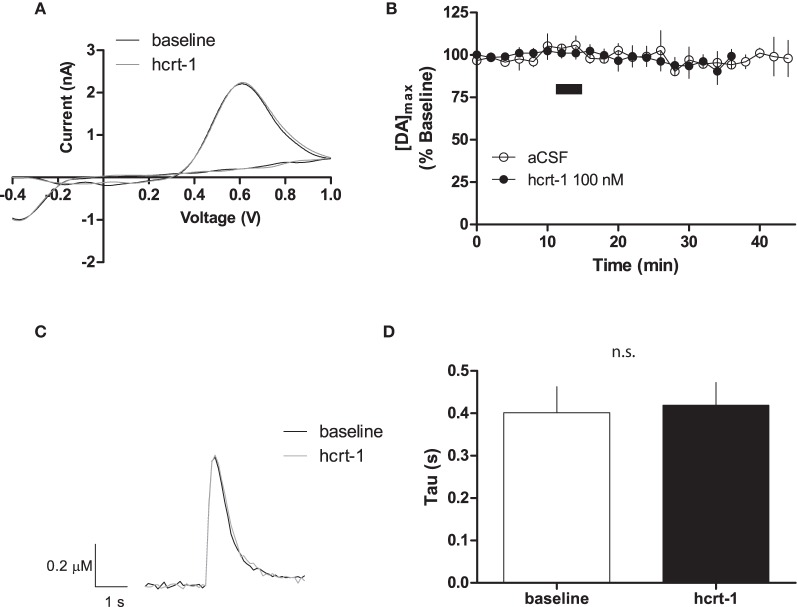
**Hcrt-1 does not modify single pulse dopamine in the NAcSh.** [DA]_o_ was electrically evoked using a single pulse in slices containing the NAcSh. **(A)** An example voltammogram from a single experiment for electrically evoked [DA]_o_ before (black line) and immediately after a 5 min hcrt-1 (100 nM, grey line) application. **(B)** Bath application of hcrt-1 (100 nM, 5 min, filled circles) did not modify evoked [DA]_o_ in NAcSh slices (*n* = 6) compared to control slices (*n* = 5, open circles) (*P* < 0.05). **(C)** A representative current-time plot from a single experiment showing [DA]_o_ evoked before (open black line) and 15 min after (grey line) application of hcrt-1 (100 nM). **(D)** The signal decay was fit with a one phase exponential curve to determine the rate of decay 5 min before and 15 min after immediately after hcrt-1 application. The rate of decay, Tau (τ), before and after hcrt-1 application was not significantly different (*P* > 0.05, *n* = 6). Bars represent mean and SEM.

To determine if hcrt-1 could modulate [DA]_o_ at dopaminergic terminals in the NAcSh where there is significant density of hcrt-immunopositive axonal fibers (Baldo et al., 2003), we applied hcrt-1 (100 nM) for 5 min to slices while evoking dopamine with a single pulse every 2 min. Hcrt-1 did not significantly alter the oxidation potential of dopamine (Figure 1A) or the maximal release of dopamine (Figure 1B; baseline: 101 ± 3% vs. 15 min after hcrt-1: 96 ± 4%, *n* = 7; *P* > 0.05). No significant change occurred in evoked [DA]_o_ over the duration of the experiment in the absence of hcrt-1 (Figure 1B; baseline: 96 ± 3% vs: 15 min after vehicle aCSF application: 95 ± 7%, *n* = 5; *P* > 0.05). The decay of the current, represented by tau (τ), has been demonstrated to be positively correlated with K_m_, suggesting that τ is an appropriate measurement of dopamine uptake (Yorgason et al., 2011). Averaged current-time plots of evoked [DA]_o_ indicated that hcrt-1 did not significantly modify dopamine uptake under single pulse conditions (Figures 1C,D, τ_baseline_ = 0.41 ± 0.05 s, τ_hcrt−1_ = 0.43 ± 0.05 s; *n* = 7; *P* > 0.05). Decay of evoked dopamine did not significantly change over the course of the experiment in the absence of hcrt-1 (τ_baseline_ = 0.44 ± 0.09 s, τ_aCSF_ = 0.41 ± 0.07 s; *n* = 5; *P* > 0.05).

### Hcrt-1 increases phasic [DA]_o_ at terminals

**Figure 2:**
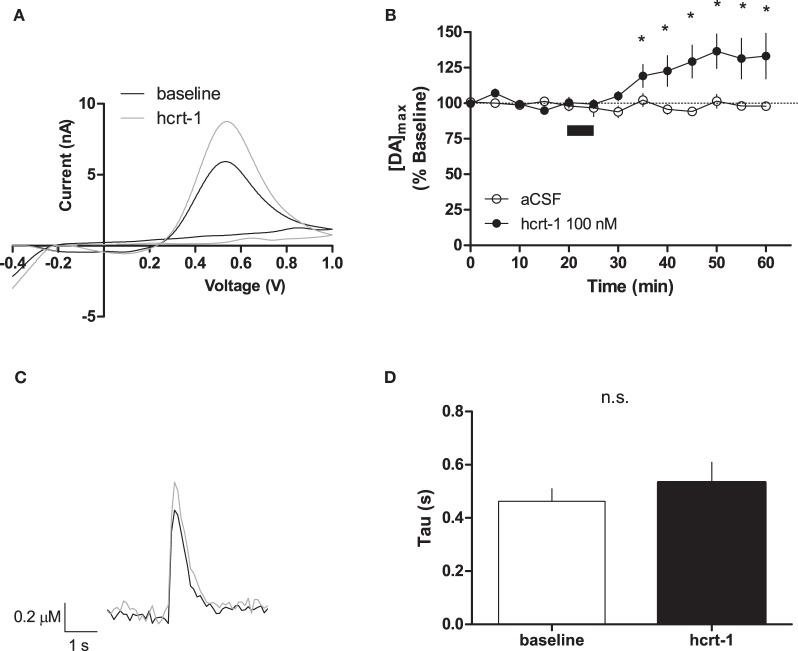
**Hcrt-1 increases phasic dopamine in the NAcSh.** Phasic [DA]_o_ was electrically evoked using 100 Hz, 5 pulses in slices containing the NAcSh. **(A)** An example voltammogram from a single experiment for electrically evoked [DA]_o_ before (black line) and immediately after a 5 min hcrt-1 (100 nM, grey line) application. **(B)** Bath application of hcrt-1 (100 nM, 5 min, filled circles) significantly increased evoked [DA]_o_ in NAcSh slices (*n* = 11) compared to control slices (*n* = 6, open circles) (^*^P < 0.05). **(C)** A representative current-time plot from a single experiment showing [DA]_o_ evoked before (open black line) and 25 min after (grey line) application of hcrt-1 (100 nM). **(D)** The signal decay was fit with a one phase exponential curve to determine the rate of decay 5 min before and 25 min after immediately after hcrt-1 application. Tau before and after hcrt-1 application was not significantly different (*P* > 0.05, *n* = 11). Bars represent mean and SEM.

Phasic dopamine release accompanies reward-predicting stimuli (Phillips et al., 2003; Roitman et al., 2004; Day et al., 2007). To test if hcrt-1 was able to modulate terminal [DA]_o_ under phasic conditions, we evoked dopamine release every 5 min with 100 Hz, 5 pulses, a parameter which increases dopamine concentration relative to a single pulse (Rice and Cragg, 2004). As reported previously, we observed an 5.5 fold increase in [DA]_o_ with phasic stimulation (4.2 ± 1.6 μM, *n* = 13) compared to a single pulse (0.8 ± 0.2 μM, *n* = 6; Patel et al., 1992; Rice and Cragg, 2004). Interestingly, hcrt-1 increased [DA]_o_ in NAcSh slices with a slow time course, reaching a maximum at 25 min after hcrt-1 application (Figures 2A,B,C, baseline: 99 ± 2% vs. 25 min after hcrt-1: 137 ± 12%, *n* = 10, *P* < 0.05). To test if peak [DA]_o_ was altered during hcrt-1 application, we evoked [DA]_o_ 2.5 min after initiation of the 5 min bath application. Surprisingly, there was no significant increase in phasically evoked [DA]_o_ immediately after hcrt-1 application (baseline: 99 ± 4 vs. hcrt-1: 108 ± 5%, *n* = 5, *P* > 0.05). To determine if the increase in [DA]_o_ was due to a change in uptake, we measured the decay of evoked DA under phasic conditions. There was no significant difference in τ 25 min after application of hcrt-1 (Figure 2D, τ_baseline_ = 0.46 ± 0.05 s, τ_hcrt−1_ = 0.53 ± 0.07 s; *n* = 10; *P* > 0.05). Taken together, these results suggest that hcrt-1 increases dopamine release only under phasic release conditions.

**Figure 3:**
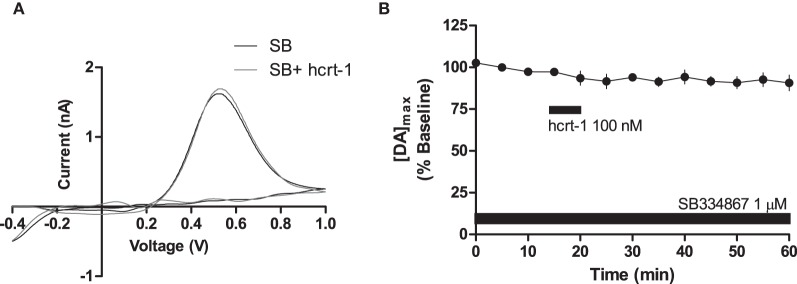
**Hcrt-1 modulation of phasic dopamine in the NAcSh is mediated by hcrt-R1.** Phasic [DA]_o_ was electrically evoked using 100 Hz, 5 pulses in slices containing the NAcSh. Slices were preincubated with SB 334967 (1 μM) for 30 min prior to application of hcrt-1 and throughout the experiment. **(A)** An example voltammogram from a single experiment for electrically evoked [DA]_o_ before (black line) and 25 min after a 5 min hcrt-1 (100 nM, grey line) application. **(B)** In the presence of SB 334867, bath application of hcrt-1 (100 nM, 5 min) did not significantly alter evoked [DA]_o_ in NAcSh slices (*n* = 4; *P* > 0.05).

To determine if hcrt-1-induced potentiation of striatal [DA]_o_ required activation of hcrt-R1, we applied hcrt-1 in the presence of the hcrt-R1 antagonist, SB334867 (1 μM) while evoking [DA]_o_ under phasic conditions. In slices preincubated for 30 min with SB334867, hcrt-1 did not alter phasically evoked [DA]_o_ (Figures 3A,B, immediately prior to hcrt-1: 97 ± 2%; 25 min after hcrt-1 application: 94 ± 3%, *n* = 4, *P* > 0.05), suggesting that striatal hcrt-R1s are required for hcrt-1-mediated potentiation of [DA]_o_.

**Figure 4:**
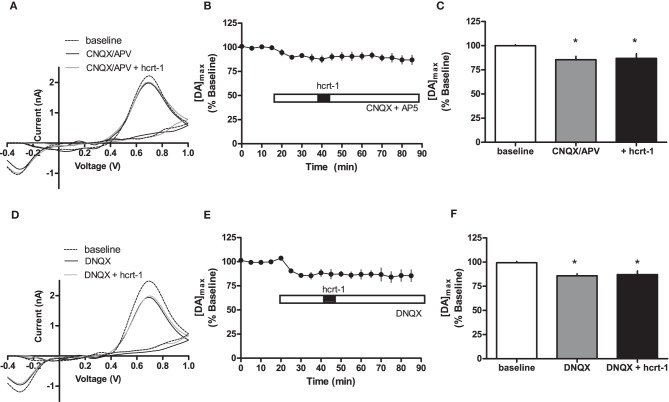
**Hcrt-1 modulation of phasic dopamine in the NAcSh requires glutamatergic signaling.** Phasic [DA]_o_ was electrically evoked using 100 Hz, 5 pulses in slices containing the NAcSh. **(A)** CNQX (10 μM) and AP5 (50 μM) were bath applied to slices for 20 min prior to application of hcrt-1 and throughout the experiment. An example voltammogram from a single experiment for electrically evoked [DA]_o_ before (hatched line), during CNQX and AP5 treatment (black line) and 25 min after a 5 min hcrt-1 (100 nM, grey line) application. **(B)** CNQX and AP5 significantly reduced [DA]_o_ in NAcSh slices (*P* < 0.05). In the presence of CNQX and AP5, bath application of hcrt-1 (100 nM, 5 min) did not significantly alter evoked [DA]_o_ in NAcSh slices (*n* = 7; *P* > 0.05). **(C)** A bargraph demonstrating maximal effects relative to baseline (open bar) of CNQX and AP5 treatment with (filled bar) or without hcrt-1 (shaded bar). CNQX and AP5 with or without hcrt-1 was significantly different from baseline (repeated measures ANOVA with a tukey's *post hoc* test (*n* = 7; *P* < 0.05). **(D)** An example voltammogram from a single experiment for electrically evoked [DA]_o_ before (hatch line), during DNQX treatment (black line) and 25 min after a 5 min hcrt-1 (100 nM, grey line) application. **(E)** DNQX significantly reduced [DA]_o_ in NAcSh slices (*P* < 0.05). In the presence of DNQX, bath application of hcrt-1 (100 nM, 5 min) did not significantly alter evoked [DA]_o_ in NAcSh slices (*n* = 5; *P* > 0.05). **(F)** A bar graph demonstrating maximal effects relative to baseline (open bar) of DNQX treatment with (filled bar) or without hcrt-1 (shaded bar). DNQX with or without hcrt-1 was significantly different from baseline (repeated measures ANOVA with a tukey's *post hoc* test (*n* = 5; *P* < 0.05). Bars represent means and SEM. ^*^p < 0.05.

Interestingly, hcrt-1 modulation of dopamine release occurs only under phasic conditions and not single pulse, suggesting that hcrt-1 may not be directly modulating dopamine terminals to promote release. One possibility is that hcrt-1 could be acting at its receptors on glutamatergic terminals within the NAcSh to increase dopamine release indirectly. To test the contribution of glutamatergic signaling to hcrt-1 modulation of [DA]_o_ in the NAcSh, we repeated the experiment in the presence of CNQX (10 μM) and AP-5 (50 μM) to block α-amino-3-hydroxy-5-methylisoxazole-4-propionate (AMPA)/Kainate and NMDA receptors, respectively. Application of excitatory amino acid receptor antagonists significantly attenuated phasically evoked dopamine by 12 ± 4% (Figures 4A,B,C; baseline: 100 ± 2% vs. CNQX/AP-5: 89 ± 4%, *n* = 6, *P* < 0.05). The effect of hcrt-1 on [DA]_o_ was abolished in the presence of CNQX and AP-5 (Figures 4A,B,C, 25 min after hcrt-1 application: 91 ± 4%, *n* = 6, *P* > 0.05). Application of the AMPA/Kainate receptor antagonist, DNQX, inhibited phasically evoked dopamine by 14 ± 2% (Figures 4D,E,F; baseline: 100 ± 1% vs. DNQX: 85 ± 4%, *n* = 5, *P* < 0.05). The effect of hcrt-1 on [DA]_o_ was also abolished in the presence of DNQX (Figures 4D,E,F; 25 min after hcrt-1 application: 87 ± 5%, *n* = 5, *P* > 0.05). Taken together, these data suggest that hcrt-1 activation of glutamatergic inputs is required for hcrt-1-mediated increase in dopamine release under phasic conditions.

## Discussion

While several studies have demonstrated that hcrt-1 administered into the VTA can increase dopamine concentration in the NAcSh (Vittoz et al., 2008), NAc core (España et al., 2011) or both (Narita et al., 2006), our data suggests an additional mechanism by which hcrt-1 can modulate dopamine concentrations in the NAcSh. We demonstrate that hcrt-1 promotes local dopamine release in NAcSh slices, suggesting that direct projections of hcrt neurons from the lateral hypothalamus can modulate dopamine release onto medium spiny neurons of the NAcSh. Notably hcrt-1-induced increase of [DA]_o_ under phasic conditions reached a maximum 15 after hcrt-1 application. This delayed effect is likely due to the necessity for the 33 amino acid peptide to penetrate the tissue to reach its receptors. Interestingly, hcrt-1 modulates NMDA receptors on VTA dopamine neurons with a similar time course (Borgland et al., 2006). Hcrt-1 potentiation of local [DA]_o_ was due to an increase in dopamine release as opposed to modulation of reuptake mechanisms as there was no effect of hcrt-1 on the decay of evoked dopamine. Taken together, in addition to hcrt-1 acting at the VTA to increase dopamine concentration in the NAc, hcrt-1 can also act at terminals to modulate release.

We observed that hcrt-1 modulated evoked [DA]_o_ under phasic conditions, but not after a single pulse repeated every 2 min. Therefore, it is likely that hcrt-1 is acting indirectly in the NAcSh to increase terminal dopamine release. One possibility is that hcrt-1 modulates GABAergic activity onto dopamine terminals in the NAcSh. While it has not been demonstrated how dopamine concentration is modulated by GABA in the NAcSh, GABA is reported to increase [DA]_o_ in the dorsal striatum (Avshalumov et al., 2003). Alternatively, hcrt-1 may act on glutamatergic inputs in the NAcSh to modulate [DA]_o_. Consistent with this idea, we demonstrated that application of AMPA or NMDA receptor antagonists inhibited a hcrt-1-induced increase of [DA]_o_.

Dopamine spillover from dopaminergic synapses onto MSNs not only activates receptors on MSNs several micrometers away (Garris et al., 1994; Gonon, 1997; Gonon et al., 2000), but may also activate presynaptic dopamine receptors on neighboring glutamatergic afferents (Wang and Pickel, 2002). Furthermore, glutamate released from excitatory inputs in the NAc can also modulate dopamine release, although its effects are complicated by the heterogeneity of expression and location of ionotropic and metabotropic glutamate receptors. There is a large body of evidence suggesting that glutamate is excitatory toward dopamine release (Cheramy et al., 1986; Clow and Jhamandas, 1988; Kalivas et al., 1989; Leviel et al., 1990; Carrozza et al., 1992; Desce et al., 1992; Jin and Fredholm, 1997; Borland and Michael, 2004). However, these studies mainly employ use of microdialysis techniques or dopamine release from isolated cells or synaptosomes. Using FSCV in NAcSh slices, we demonstrate that application of ionotropic glutamate receptor antagonists, AP-5 and CNQX or DNQX alone, significantly inhibited phasically evoked [DA]_o_. In contrast to the present study, phasically evoked dopamine from dorsal striatum slices was increased in the presence of the AMPA receptor antagonist, GYKI-52466 (Avshalumov et al., 2003, 2008), the NMDA receptor antagonist, AP-5 or the broad spectrum ionotropic glutamate receptor antagonist, kynurenate (Wu et al., 2000). Moreover, glutamate spillover can activate metabotropic glutamate receptors to inhibit evoked dopamine (Zhang and Sulzer, 2003). A possible reason for the discrepancy between our study and the other studies employing FSCV in slices is that the distribution of ionotropic receptors and glutamatergic inputs may differ between dorsal striatum and NAcSh (Pennartz et al., 1994; Sesack and Grace, 2010). Indeed, NMDA or AMPA receptors increase dopamine efflux in a study employing *in vivo* voltammetry in the NAc (Svensson et al., 1994) and that DNQX can inhibit quisqualate evoked dopamine concentration in a study employing microdialysis in the NAc (Imperato et al., 1990).

Hcrt-1 acts with similar efficacy at both hcrt-R1 and hcrt-R2 (Sakurai et al., 1998). Because hcrt-1 has been implicated in addiction-related behaviors and modulation of dopamine via its action in the VTA, we were interested in testing if this peptide altered local dopamine release at axon terminals. Interestingly, the hcrt-1-mediated increase in NAcSh dopamine was inhibited by SB 334867, suggesting this effect was mediated by hcrt-R1. Hcrt-R2, and to a lesser extent hcrt-R1 are expressed within the NAcSh (Trivedi et al., 1998; Marcus et al., 2001; Martin et al., 2002). Notably, cell bodies of glutamatergic neuron that project to the NAcSh, including the prefrontal cortex and basolateral amydala, express hcrt-R1 (Trivedi et al., 1998; Lu et al., 2000; Marcus et al., 2001) and it is possible that their axon terminals may also express hcrt-R1. Furthermore, hcrt-1 promotion of glutamate release has been demonstrated in other brain regions such as the VTA (Borgland et al., 2006, 2009; Wang et al., 2009), amygdala (John et al., 2003) and hippocampus (Stanley and Fadel, 2011). Therefore, it is feasible that activation of hcrt-R1s on glutamatergic inputs in the NAcSh can increase glutamate release. Our results support the possibility that hcrt-R1 activation on glutamatergic inputs can promote dopamine release in the NAcSh.

Dense staining of hcrt terminals in the NAcSh has been observed (Peyron et al., 1998; Baldo et al., 2003), suggesting that hcrt-1 has direct action in the NAc. Local action of hcrt-1 at dopaminergic terminals may potentiate effects of hcrt-1-mediated dopamine release via its action in the VTA. Indeed, other studies have demonstrated that application of hcrt-1 or hcrt-2 into the NAcSh can potentiate dopaminergic activity. For example, in NAcSh slices, when hcrt-2 was co-applied with dopamine, its effect on firing rate was significantly potentiated in 2/3 of MSNs when compared to hcrt-2-induced or dopamine-induced firing alone (Mori et al., 2011). Another study demonstrated that hcrt-1 or hcrt-2 administered directly in the NAcSh potentiated the effects of dopamine receptor agonists on contraversive pivoting behavior (Kotani et al., 2008). Thus, consistent with our study, it is likely that an interaction between hcrt and dopaminergic systems for reward seeking behavior may occur in the NAcSh. Furthermore, one can speculate that local action of hcrt-1 in the NAcSh may potentiate dopaminergic responses mediated by hcrt-1 or other drug action in the VTA. In summary, hcrt-1 increased phasically evoked [DA]_o_ in the NAcSh. This effect required activation of AMPA receptors and hcrt-R1. This study demonstrates an additional mechanism by which hcrt-1 can modulate dopamine release and potentially influence appetitive behaviors.

### Conflict of interest statement

The authors declare that the research was conducted in the absence of any commercial or financial relationships that could be construed as a potential conflict of interest.
